# Blood Signature of Pre-Heart Failure: A Microarrays Study

**DOI:** 10.1371/journal.pone.0020414

**Published:** 2011-06-24

**Authors:** Fatima Smih, Franck Desmoulin, Matthieu Berry, Annie Turkieh, Romain Harmancey, Jason Iacovoni, Charlotte Trouillet, Clement Delmas, Atul Pathak, Olivier Lairez, François Koukoui, Pierre Massabuau, Jean Ferrieres, Michel Galinier, Philippe Rouet

**Affiliations:** 1 INSERM/Universite Paul Sabatier UMR 1048, Institut des Maladies Métaboliques et Cardiovasculaires (I2MC), Toulouse, France; 2 Rangueil Hospital University, Cardiology Department, Toulouse, France; University of Chicago, United States of America

## Abstract

**Background:**

The preclinical stage of systolic heart failure (HF), known as asymptomatic left ventricular dysfunction (ALVD), is diagnosed only by echocardiography, frequent in the general population and leads to a high risk of developing severe HF. Large scale screening for ALVD is a difficult task and represents a major unmet clinical challenge that requires the determination of ALVD biomarkers.

**Methodology/Principal Findings:**

294 individuals were screened by echocardiography. We identified 9 ALVD cases out of 128 subjects with cardiovascular risk factors. White blood cell gene expression profiling was performed using pangenomic microarrays. Data were analyzed using principal component analysis (PCA) and Significant Analysis of Microarrays (SAM). To build an ALVD classifier model, we used the nearest centroid classification method (NCCM) with the ClaNC software package. Classification performance was determined using the leave-one-out cross-validation method. Blood transcriptome analysis provided a specific molecular signature for ALVD which defined a model based on 7 genes capable of discriminating ALVD cases. Analysis of an ALVD patients validation group demonstrated that these genes are accurate diagnostic predictors for ALVD with 87% accuracy and 100% precision. Furthermore, Receiver Operating Characteristic curves of expression levels confirmed that 6 out of 7 genes discriminate for left ventricular dysfunction classification.

**Conclusions/Significance:**

These targets could serve to enhance the ability to efficiently detect ALVD by general care practitioners to facilitate preemptive initiation of medical treatment preventing the development of HF.

## Introduction

The risk for developing heart failure (HF) in Western countries is estimated to be 33%, with a 8 year post-diagnosis mortality rate of 75% [1] and the annual cost of treatment in the US was estimated at $37.2 billion in 2009 [1]. Epidemiologic studies have demonstrated that cardiovascular risk factors such as hypertension, diabetes and obesity are precursors of HF [1]. These factors induce modification of the myocardium structure and lead to functional alterations of the heart [2] including a reduction in the left ventricular ejection fraction (LVEF). Identification of patients at the “pre-heart failure” stage can prevent the development of HF through the initiation of adapted medical and non-medical strategies. This silent preclinical state (pre-heart failure) is referred as asymptomatic left ventricular dysfunction (ALVD) and can only be diagnosed by transthoracic echocardiography. ALVD, common in the general population, leads to a high risk of developing overt HF. Indeed, compared to individuals with normal LVEF, ALVD subjects have a 12-fold increase in the annual rate of hospitalization for first-event HF [3] and a 4-fold increase in the risk of death over a 6-year period [2]. Effective large-scale screening for ALVD, at present a difficult task representing a major unmet clinical challenge, requires a determination of ALVD biomarkers.

Despite the fact that screening for ALVD has been advocated for over a decade [3], [4], there are no ALVD biomarkers. Indeed, ALVD diagnosis requires a sophisticated echocardiographic analysis, which is both time-consuming and costly, and is not applicable to the large population of individuals at risk. The lack of biomarker(s) is of importance because ALVD is highly prevalent due to the general increase in cardiovascular risk factors [5]. ALVD has become established as a predictive early indicator of severe HF [6]. Follow-up studies have shown that ALVD subjects display an average annual chronic heart failure rate of 4.9 to 20%, with a mortality rate of 5.1 to 10.5% [7], [8]. Such observations were recently confirmed in a 5-year survival rate analysis that showed a death rate of 31% for subjects suffering from ALVD and of 47% for patients with systolic HF [9]. Finally, the SOLVD study demonstrated that the treatment of ALVD results in a significantly delayed occurrence of HF [10]. Therefore, it is of clinical relevance to identify ALVD individuals in the general population before they develop overt HF.

The objective of the present study was to evaluate the impact of ALVD on the human transcriptome and to identify a specific molecular signature based on differential gene expression. Ideally, the molecular signature should be independent of cardiovascular risk factors (such as hypertension, diabetes, obesity, dyslipidemia…) and should be useful to sort ALVD individuals among subjects with cardiovascular risk factors. Moreover, the transcriptome analysis allowed us to perform a global analysis without prior knowledge or hypothesis of the gene whose expression could be affected by the disease.

We analyzed white blood cell transcriptomes since gene expression patterns in peripheral blood has been validated in humans [11] as a basis for the detection and diagnosis of diseases such as chronic [12], [13] and acute heart failure [14]. Indeed, the blood is a dynamic and interactive tissue that communicates with all cells of the body and can therefore display perturbations indicative of disease. Previous work have shown that blood cells share 84% of their transcriptome with the heart [15] and that some gene regulations in blood are similar to other organs such as the heart [13], [14]. Thus, peripheral blood is likely to become a useful resource in the diagnosis of systemic diseases, selection of treatment methods and disease outcome prediction [15]. In this work we show that white blood cells retain information of ALVD and provide a set of genes that could be used to pre-screen patients for ALVD before time-consuming echocardiographic confirmation of the disease.

## Results

### Patient Inclusions

**Figure 1:**
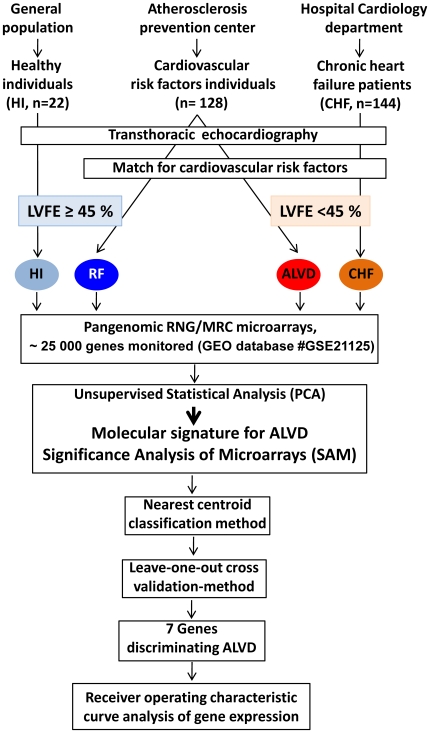
Flow chart of recruitment protocol involving 294 subjects and overall study design. Healthy volunteers (HI) were recruited from the general population, individuals with cardiovascular risk factors (RF) were from the atherosclerosis prevention center and patients with chronic heart failure (CHF) were recruited from the cardiology department at Rangueil Hospital, Toulouse. All subjects underwent transthoracic echocardiography for left ventricular ejection fraction (LVEF) assessment. We used a threshold value of LVEF<45% to sort individuals into 4 groups: HI (light blue), RF with LVEF≥45% (dark blue), ALVD (red), and CHF (orange) with LVEF<45%. We identified 9 ALVD cases out of the 128 subjects tested with cardiovascular risk factors. We used the set of cardiovascular risk factors (age, gender, arterial hypertension, diabetes, obesity, dyslipidemia and heredity) based on the characteristics of the ALVD subjects to match the study groups (n = 9). White blood cell gene expression profiling was performed using pangenomic microarrays for all 4 groups. Data were statically analyzed using unsupervised primary component analysis (PCA) and by Significance Analysis of Microarrays (SAM software) which defined the false discovery rate. Then to build an ALVD classifier model, we used the nearest centroid classification method (NCCM) with the ClaNC software package. Classification performance was determined using the leave-one-out cross-validation method. Expression levels of 7 genes capable of discriminating ALVD were compared between the 4 groups and each gene's capability to discriminate patients with LVEF<45% was evaluated using Receiver Operating Characteristic (ROC) analysis.

All the 294 subjects underwent transthoracic echocardiography for left ventricular ejection fraction (LVEF) assessment (**Fig. 1**). Healthy volunteers (HI) without cardiovascular risk or echocardiographic abnormalities were recruited from the general population. Individuals with cardiovascular risk factors and normal left ventricular ejection fraction (RF) and individuals with cardiovascular risk factors and asymptomatic abnormal left ventricular ejection fraction (ALVD) were from the atherosclerosis prevention center. Patients with stable systolic chronic heart failure (CHF) were recruited from the cardiology department at Rangueil Hospital, Toulouse. We used a threshold value of LVEF<45% to sort individuals into 4 groups: HI, RF with LVEF≥45%, ALVD, and CHF with LVEF<45%. We identified 9 ALVD cases out of the 128 subjects tested with cardiovascular risk factors. The set of cardiovascular risk factors used to match the study groups were determined based on the characteristics of the ALVD subjects (n = 9): mean age 58 years old, 78% male, 44% hypertensive, 33% diabetes, 44% obesity, 56% dyslipidemia and 22% heredity. We defined two comparative groups: 1- (RF) individuals with cardiovascular risk factors and normal left ventricular ejection fraction (62<LVEF<81%); 2- (ALVD) individuals with cardiovascular risk factors and systolic ALVD (32<LVEF≤44%). Two additional groups were used as controls: 3- (HI, negative control) Healthy volunteers without cardiovascular risk or echocardiographic abnormalities and 4- (CHF, positive control) patients with chronic stable systolic heart failure (18<LVEF<44%). Moreover, all individuals were extensively phenotyped to check for clinical and biochemical parameters. Medications and biochemical data were not significantly different between comparative groups (RF and ALVD) (**Table 1**).

**Table 1:** Cardiovascular risk factors, clinical and biochemical parameters of study groups.

Groups	HI	RF	ALVD	CHF
Age (years)	55 (72-45)	55 (69-38)	58 (84-31)	55 (83-23)
Male %	78	78	78	67
Hypertensive %	0	44	44	44
Diabetes %	0	33	33	33
Obesity %	0	44	44	56
Dyslipidemia %	0	56	56	44
Heredity %	0	33	22	11
BMI	24±3	28±3	29±4	30±5
Systolic blood pressure (mm Hg)	128±16	138±12	123±17	124±19
Diastolic blood pressure (mm Hg)	81±9	80±14	75±12	76±9
Heart rate	64 (45-86)	64 (57-72)	70 (54-87)	78 (60-102)
**Medications**				
Statines %	0	22	55	44
β Blockers %	0	11	44	78 =
ARBs or ACEIs %	0	48	55	88 =
**Labs (means ± s.d.)**				
BNP (pg/ml)	11±9	15±10	27±23	164±151$
Na+ (mM)	140±1	140±1	139±2	138±4
creatinine (µM)	80±6	83±9	96±23	126±73
Hb (g/dl)	14.7±1.1	14.3±1.1	14.1±1.8	13.4±1.8
Leukocytes (cells/µl)	5735±1225	6159±1433	6765±1533	8571±2734
Lymphocytes (cells/µl)	1865±412	1988±428	1918±499	1898±677
Neutrophils (cells/µl)	3498±898	3630±1073	4107±1153	6049±2619
**Echocardiography**				
LVEF %	73 (65-81)	71 (62-81)*	39 (32-44)	33 (18-44)
LVEDD (mm)	48 (53-44)	49 (53-42)*	61 (66-48)	61 (64-56)
Shortening fraction %	39 (44-30)	37 (45-28)*	23 (27-18)	20 (35-8)

Proportion of individuals with the indicated risk factor for each of the 4 groups of the study are indicated as percentages or averaged value:- HI, Healthy volunteers without cardiovascular risk or echocardiographic abnormalities; RF, individuals with cardiovascular risk factors and normal left ventricular ejection fraction (LVEF); ALVD, asymptomatic left ventricular dysfunction individuals with cardiovascular risk factors and abnormal left ventricular ejection fraction; CHF, Patients with chronic stable systolic heart failure. All individuals were extensively phenotyped to check for clinical and biochemical parameters. Family history of coronary artery disease is defined as a family history of coronary event before 55 years in men and/or 65 years in women occurring in first degree relatives.

*indicates *P*<0.05 for statistical comparison between RF and ALVD groups.

$ indicates *P*<0.05 for statistical comparison between ALVD and CHF groups. BNP, B-type natriuretic peptide. ARBs: angiotensin receptor blockers. ACEIs : angiotensin converting enzyme inhibitors.

= indicates P<0.05 for statistical comparison between groups. LVEDD : left ventricular end-diastolic diameter. LVESD : left ventricular end-systolic diameter. Shortening fraction % = (LVEDD−LVESD)/LVEDD×100.

### BNP Measurements

Healthy individuals had plasma BNP levels within the range 11±9 pg/ml. Plasma BNP levels were not statistically different between ALVD (27±23 pg/ml) and RF (15±10 pg/ml) patients, but were significantly increased in CHF (164±151 pg/ml) (**Table 1**).

### ALVD predictive model based on white blood cell transcriptome

**Figure 2:**
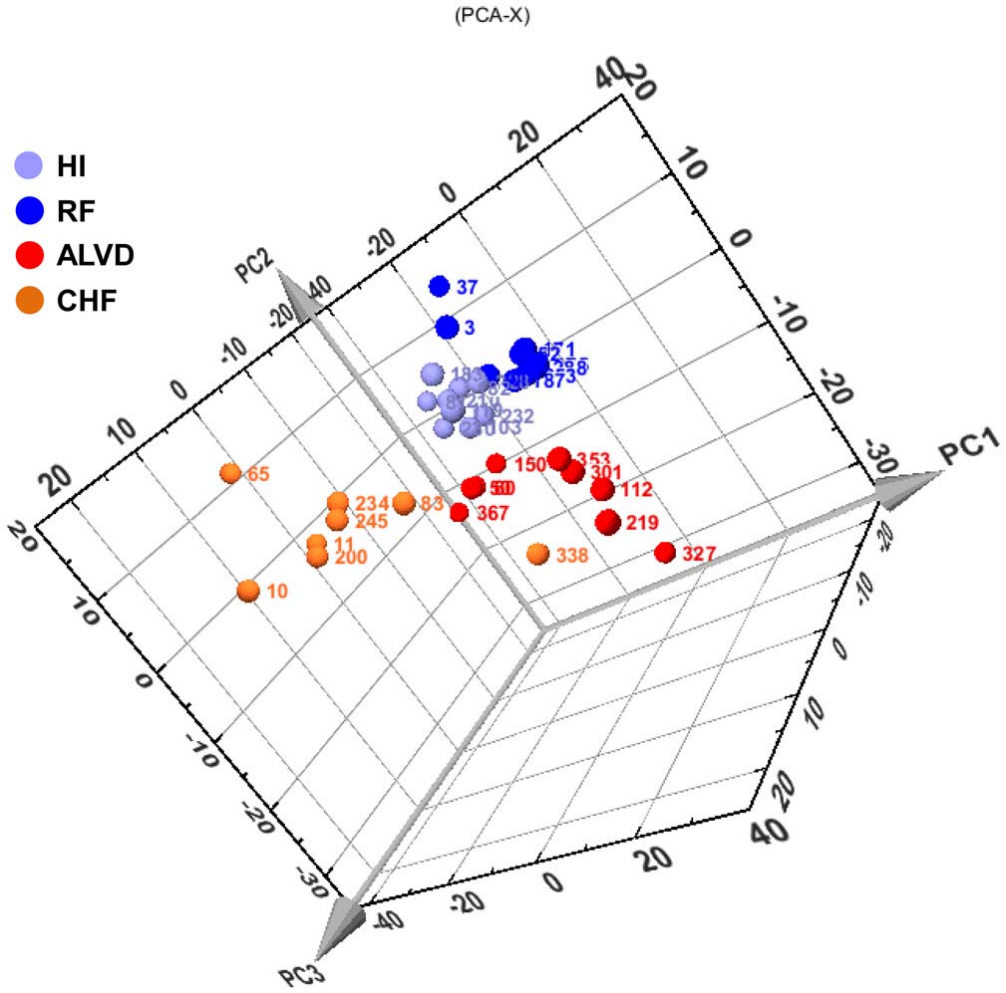
Unsupervised primary component statistical analysis (PCA) of blood transcriptome data reveals a molecular signature for ALVD. Three dimensional plot of the three first components(PC1, PC2, PC3) of the blood gene expression data from healthy subjects (HI, light blue), cardiovascular risk factor individuals (RF, dark blue), individuals with asymptomatic left ventricular dysfunction groups (ALVD, red) and chronic heart failure patients (CHF, orange). These three components can classify subjects according to their group and distribute the subjects in grouped locations in the defined space. Numbers in colors indicate subjects' identities. The relative expression level used is defined by the ratio obtained with the tested sample to the signal obtained using the common reference, an equimolar mix of all the RNA used to generate a reference signal.

We used the RNG-MRC 25k human pangenomic glass microarrays from the National Genopole Network to analyze blood gene expression of 25,341 genes. An unsupervised PCA analysis of the expression data was able to cluster patients into their respective groups: HI, RF, ALVD and CHF (**Fig. 2**) and revealed that blood gene expression profiles provide a molecular signature characteristic of ALVD. In order to build an ALVD predictive model, we used the nearest centroid classification method (NCCM) from the ClaNC software package [16]. NCCM provided a set of genes whose expression profile led to a 100% successful classification of ALVD patients out of the 4 groups of individuals. We further tested the strength of our model of gene expression-based group prediction by leave-one-out cross-validation method [17]. The classifier model accuracy and precision computed from the confusion matrix (**Table 2**) were 83% and 78%, respectively. The Fisher's exact test *P*-value (n = 18, P = 0.015) pointed out the robustness of the model.

**Table 2:** Leave-one-out's confusion matrix.

	ALVD Individuals	RF Individuals
Classified as LVEF<45	(true positive)78%	(false positive)11%
Classified as LVEF≥45%	(false negative)22%	(true negative)89%

Classification performance of the nearest centroid classifier was determined using leave-one-out cross-validation method. Calculation of the classifier model accuracy and precision by standard formulae provided 83% and 78%, respectively. Fisher's exact test *P*-value (n = 18) *P* = 0.015.

### Additional ALVD patient group validation

In addition to our original patient cohort, we obtained blood samples from 8 additional ALVD and 8 RF individuals not matched for cardiovascular risk factors. These additional subjects fulfilled the ALVD parameter definition as they were in NYHA I class with an EF<45%, lacked HF symptoms, had low plasma BNP levels (25±11 pg/ml) which was not significantly different from the initial ALVD group (P = 0.87) (**Table 3**). White blood cell gene expression analysis further validated using the ClaNC ALVD predictive model which gives 75% of true positive rate and 100% of true negative rate. Thus, the accuracy and precision of the prediction were 87% and 100%, respectively (Fisher's exact test *P*-value = 0.007; n = 16).

**Table 3:** Cardiovascular risk factors, clinical and biochemical parameters of the ALVD validation group (n = 8) and RF individuals (n = 8).

Groups	ALVD Individuals	RF
**LVEF %**	39 (20-45)	74 (65-81)*
**Age (years)**	63 (75-40)	67 (83-55)
**Male %**	87	100
**Hypertensive %**	62	37
**Diabetes %**	12	25
**Obesity %**	0	12
**Dyslipidemia %**	50	37
**Heredity %**	-	0
**BMI**	25±3	28±5
**Systolic blood pressure (mm Hg)**	144±19	145±12
**Diastolic blood pressure (mm Hg)**	82±10	86±8
**Labs (mean ± s.d.)**		
**BNP (pg/ml)**	25±11	14±9
**Na+ (mM)**	140±2	140±1
**creatinine (µM)**	110±38	92±12
**Hb (g/dl)**	14.0±0.9	14.4±0.4
**Leukocytes (cells/µl)**	6043±1041	6682±1360
**Lymphocytes (cells/µl)**	1433±332	1959±700
**Neutrophils (cells/µl)**	3975±744	3049±668

*indicates *P*<0.05 for statistical comparison between RF and ALVD validation groups.

### Discriminant genes

ClaNC defined a set of discriminant genes for the ALVD group including ALK, SLC43A2, NGFB, FBXW7, TMEM79, UBN1 and FECH (**Table 4**). SAM software analysis revealed that these genes were significantly differentially expressed between RF and ALVD with a false discovery rate ranging between 1.4 and 3.2 which is below 5%, a value generally used in transcriptome studies and is indicative of good reliability of the differential expression of these genes. Moreover, differential expression of these 7 genes in white blood cells from RF and ALVD groups was confirmed by realtime qPCR (**Figure S1**). Using human heart samples (right appendage) from patients undergoing coronary by-pass during heart surgery, we observed a similar regulation (induction or repression) that was statistically significant for 3 genes (UBN1, NGF, FECH) when comparing gene expression levels in the no HF *vs* the CHF group. ALK expression displayed a tendency to be increased in heart from heart failure patients but the difference was not statistically significant (p = 0.15) (**Figure S2**).

**Table 4:** ClaNC defined set of 7 discriminant genes for ALVD.

Gene symbol	Ensembl accession number	Full gene name	Protein location
ALK	ENSG00000171094	Anaplastic lymphoma receptor tyrosine kinase	plasma membrane
SLC43A2	ENSG00000167703	Solute carrier family 43, member 2	plasma membrane
NGFB	ENSG00000134259	Nerve growth factor (beta polypeptide)	Extracellular space
FBXW7	ENSG00000109670	F-box and WD repeat domain containing 7	Nucleus; cytoplasm
TMEM79	ENSG00000163472	Transmembrane protein 79	plasma membrane
UBN1	ENSG00000118900	Ubinuclein 1	Nucleus
FECH	ENSG00000066926	Ferrochelatase	Mitochondrion

Gene symbol, Ensembl accession number, full gene name and protein location, as provided by Ingenuity's Pathway Assist software, are indicated.

Moreover, Ingenuity's Pathway Analysis revealed that three genes encoded membrane proteins: the kinase ALK, TMEM79 and SLC43A2. FECH is a mitochondrial protein and FBXW7 encodes a cytoplasmic component of E3 ubiquitin protein ligase, which regulates the proteolytic machinery. UBN1 is a transcription factor involved in the formation of senescence-associated heterochromatin foci. NGFB (Nerve Growth Factor Beta) is a pleiotropic neurotrophin discovered over 50 years ago and involved in the development, maintenance of function and regeneration of nerve cells in the heart [18].

**Figure 3:**
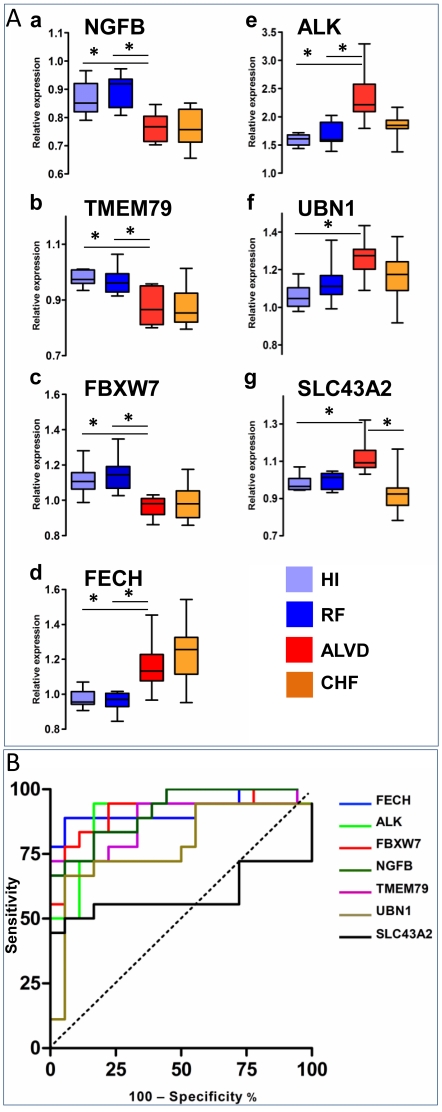
Expression levels for 7 genes discriminate the ALVD group. A. a–g Relative expression levels of the 7 genes, sorted by the nearest centroid classifier, are assessed for HI (light blue), RF (dark blue), ALVD (red) and CHF (orange) groups. The relative expression level used is defined by the ratio obtained with the tested sample to the signal obtained using the common reference, an equimolar mix of all the RNA used to generate a reference signal. The box plot presents the median, lower and upper quantiles (25^th^, 75^th^ percentiles) lower and upper whiskers represent the 10th and 90th percentiles. * *P*<0.05 where indicated, estimated by one-way ANNOVA. B. Receiver-operating characteristic (ROC) analysis of ALVD discriminant genes using HI with RF (LVEF≥45% as disease free) and ALVD with CHF (LVEF<45% as left ventricular dysfunction) groups. Area under curve (AUC), confidence interval and *P* values to find an AUC value of 0.5 (null hypothesis) for each gene are depicted in **Table 5**. ROC curves for each of the 7 genes are displayed on a single figure. With the exception of SLC43A2, ALVD discriminant genes are also CHF biomarkers *i.e.* left ventricular dysfunction biomarkers.

We next looked for the pertinence of each of these genes with respect to ALVD subject classification. Box-and-whisker plots, which depict variations in gene expression levels, showed that expression of NGFB, TMEM79 and FBXW7 was significantly down-regulated in ALVD group (**Fig. 3Aa, b, c**), whereas FECH, ALK, UBN1 and SLC43A2 expression were significantly increased in ALVD cases (**Fig. 3Ad, e, f, g**).

A series of ROC curves were created for each of the proposed discriminant genes as related to the incidence of left ventricular dysfunction (LVEF<45%) (**Fig. 3B**). Six genes out of seven (NGFB, TMEM79, FECH, FBXW7, ALK, UBN1) statistically differed from the null hypothesis (p<0.005 or p<0.0001, **Table 5**) and provided a good discrimination (no overlap in the two distributions). The area under the curve (AUC), ranged from 0.78 to 0.92 for predicting left ventricular dysfunction. Only the SLC43A2 gene harbored a weak AUC (0.59) and was a poor predictor (p = 0.35; **Table 5**).

**Table 5:** ROC curve statistical data.

Gene	AUC	95% CI	*P* value
NGFB	0.91	0.82–1.00	<0.0001
TMEM79	0.88	0.76–1.00	<0.0001
FBXW7	0.91	0.81–1.01	<0.0001
FECH	0.92	0.82–1.02	<0.0001
ALK	0.88	0.76–1.00	<0.0001
UBN1	0.78	0.62–0.94	= 0.0036
SLC43A2	0.59	0.38–0.80	= 0.3589

Area under curve (AUC), 95% confidence interval (CI) and *P* values are indicated.

## Discussion

Epidemiological echocardiographic studies, have revealed that ALVD has a prevalence of 0.9 to 12.9%, depending on the definition of the threshold value for LVEF (<30% up to <54%) and the population studied [2], [19], [20], [21], [22]. We used a threshold value of LVEF<45% to screen for truly asymptomatic subjects in a population possessing cardiovascular risk factors and found an ALVD prevalence of 7% (9 ALVD out of 128 subjects tested with cardiovascular risk factors), in line with values reported in previous works (6.7%) [2].

Natriuretic peptides, B-type natriuretic (BNP) and N-terminal-proBNP (NT-ProBNP), released from the heart in response to pressure and volume overload, have become the main biomarkers for assessing HF (reviewed in [23]). BNP was shown to be of limited use in the detection of ALVD in diabetic patients [24]. While the negative predictive value of NT-ProBNP was 96 to 100% [25], [26], its positive predictive value ranged from 6 to 33%, which limited its use in ALVD detection [25], [26]. In our study, ALVD individuals (test and validation groups together) had plasma BNP levels within the physiological range [23] : (26±18 pg/ml; n = 17). As generally observed in CHF patients, the symptomatic and positive controls of this study, BNP levels were significantly increased (164±151 pg/ml). These values, in accordance with Daniels and Maisel [23], confirm that the ALVD subjects selected in this study are truly asymptomatic individuals. Since BNP levels could not be used satisfactorily for ALVD detection within our subjects, we attempted to find new biomarkers using holistic approaches. Considering DNA microarrays are powerful tools allowing for large scale screenings, we used the RNG-MRC 25k human pangenomic glass microarrays from the National Genopole Network to analyze blood gene expression of 25,341 genes. An unsupervised Principal Component Analysis (PCA) of the global gene expression data clustered individuals into their respective groups (HI, RF, ALVD and CHF) and revealed a molecular signature characteristic of ALVD. Since it is difficult to define gene sets belonging to each clusters in most implementation of PCA [27] and in order to build an ALVD classifier model, we used the nearest centroid classification method (NCCM) which provides an accurate predictor [16]. NCCM defined a set of 7 genes whose expression profile led to the classification of ALVD subjects out of the four groups of individuals. We further tested the strength of our model of gene expression-based group prediction by leave-one-out cross validation, a technique recognized for its small bias [17], which utilizes a single observation from the original sample as the validation data and the remaining observations as the training data. The confusion matrix from the leave one-out cross-validation results, led us to conclude that the defined pool of genes efficiently classifies ALVD subjects. Moreover, the test of additional patients included in an ALVD patient validation group showed that the accuracy (87%) and the precision (100%) of the gene expression based predictive model were close to the ones determined by leave-one-out cross-validation (88 and 78% for accuracy and precision, respectively).

Interestingly, the decrease in NGFB gene expression that we observed in white blood cells was previously reported in both human and experimental heart failure [28]. Our data suggest that NGFB down-regulation could occur earlier in blood, in pre-heart failure state. Moreover, SAM analysis revealed significant overexpression EGR1 and CCR2 genes in CHF group (data not shown) in agreement with previous report [13].

UBN1, NGF and FECH genes displayed similar statistically significant regulation (up or down regulated) in hearts samples from heart failure patients (**Figure S2**) but expression of the three remaining genes (TMEM79, FBXW7 and SLC43A2) could not be quantified, probably because of their low expression in heart. This is in accordance with other studies showing that white blood cells express a large number of heart genes but not all [15] and that some genes expressions regulations in heart are also observed in white blood cells which might serve as surrogate markers for heart failure [14].

The data presented in this paper has the potential to provide tools for ALVD screening. We have chosen to match cardiovascular risk factors within comparative groups (RF and ALVD), which limited the number of blood samples used in the analysis, but also resulted in the determination of an ALVD blood transcriptome signature as well as the elucidation of putative marker genes. One advantage of this prospective monocenter study is that the microarrays and clinical data quality were homogeneous. Gene expression analysis has recently undergone a tremendous evolution and could before long be carried out in a routine clinical setting (outpatient department, general practice) which would represent a cost effective method to identify ALVD subjects and prevent HF development. We anticipate that large screen of population at risk for ALVD examinated by primary-care physician could be screened at low cost by blood microfluidic qPCR and if positive for ALVD would be addressed to echocardiography for ALVD confirmation. This strategy would identify subject in the early phase of this silent disease and prevent them from being symptomatic by early medication and appropriate lifestyle-related advices.

## Materials and Methods

### Study design and population

The present study was conducted with 294 individuals (Healthy volunteers (HI, n = 22), arteriosclerosis prevention (individuals with cardiovascular risk factors, n = 128) and cardiology departments (patients with chronic stable heart failure (CHF, n = 144) at the French Medical University Hospital of Toulouse (Fig. 1 and Table 1). All patients underwent echocardiography and left ventricular ejection fractions (LVEF) were determined by contour analysis using the two-dimensional Simpson's method [29]. According to the European guidelines [30], left ventricular systolic dysfunction was defined by a LVEF<45%. We defined two comparative groups: 1- (RF) individuals with cardiovascular risk factors and normal left ventricular ejection fraction; 2- (ALVD, n = 9) individuals with cardiovascular risk factors and systolic ALVD.

Right ventricular function was evaluated during the echocardiography examination with the tricuspid annular plane systolic excursion (TAPSE) index and/or the S-wave velocity, patients presenting a TAPSE index <16 mm and/or a S-wave <10 cm/s were excluded from the study. Two additional groups were used as controls: 3- (HI, n = 22) Healthy volunteers without cardiovascular risk or echocardiographic abnormalities and 4- (CHF, n = 144) Patients with chronic stable systolic heart failure. CHF etiology was 33% ischemia, 11% arterial hypertension, 11% valvular disease, 44% dilated idiopatic cardiomyopathy. Atrial fibrillation was found in 33% of CHF patients. Exclusion critera were presence of infarct or recent angor (<6 months), infiltrative cardiomyopathy, cerebral vascular event, kidney failure, liver failure, blood disease, ongoing cancer or cured since 5 years or less, toxicomania and alcohol abuse, psychiatric disorders and participation to a clinical trial within the last 30 days. Using SAS software, we defined groups of 9 subjects perfectly matched to the ALVD group for HF risk factors such as age, gender, arterial hypertension, diabetes, obesity, dyslipidemia and heredity. BNP was assessed using the Centaur Bayer kit (Bayer HealthCare, France) and a Centaur (Siemens, France) hospital automat as recommended by the manufacturers. This research protocol was approved by the institutional review boards and ethics committee. All participants gave written informed consent (ClinicalTrials.gov number: NCT01024049).

### Human heart samples

After ethical committee approval, all patients included in the sub-study gave their informed consent for sample collection and molecular analysis prior to their inclusion. Patients were carefully selected by the physicians from Department of Cardiology, Toulouse University hospital prior to cardiac surgery for coronary by-pass due to coronary disease. Samples from right auricle appendages were collected from the department of cardiovascular surgery of Toulouse University Hospital at the beginning of cardiac surgery and were of extra corporeal circulation. Samples were immediately washed in cold buffer, snap frozen in liquid nitrogen and maintained at −80°C until analysis. Total RNA was isolated from the myocardium by using TRIzol reagent (Invitrogen, France) as described by the manufacturer. RNA integrity was checked by capillary electrophoresis using an Experion (Biorad) apparatus.

### Microarrays Analysis

We collected blood samples in 8 ml BD CPT vacutainer tubes that were processed immediately after collection according to the manufacturer's protocol. Total RNA was purified from collected white blood cells using the RNeasy kit (Qiagen) in a Qiacube (Qiagen) automated protocol. Total RNA integrity was checked by capillary electrophoresis (Experion, Bio-Rad). Samples with RNA Quality Indicator ≥8.5/10 were selected for analysis. Total RNAs were precisely quantified using RiboGreen and a Victor™ X5 2030 multilabel reader (Perkin Elmer). Total RNA (300 ng) were used for fluorescent labeling (QuickAmp Labeling, Agilent). Fluorescent RNAs were further purified on RNeasy columns. We used a pooled reference sample as a common reference for all hybridizations [31]. Labeled RNA were hybridized to human pangenomic glass microarrays from the consortium Reseau National des Génopole France and Medical Research Council, England; consisting of ∼25,000 51-mer oligonucleotide probes. After standard hybridization, glass arrays were washed on a Ventana robotized apparatus and scanned using a GenePix 4000 scanner (Axon). Scanned images were processed by X-dot reader software with operator validation. Microarray data were deposited in the GEO database (#GSE21125) and followed MIAME requirements.

### Real time quantitative PCR analysis

Genomic DNA was removed from RNA samples by DNAfree kit according to the manufacturer protocol (Ambion). Reverse transcription was carried-out using 150 ng total RNA and Superscript III according to the manufacturer protocol (Invitrogen). Realtime quantitative PCR was performed in a MyiQ real time PCR detection system using Sybergreen and as previously described [32]. Oligos were synthetised by Eurogentec company and designed with PerlPrimer [33] and were as follow: hNGF forward, 5′-GAGGTGCATAGCGTAATGTC-3′ and reverse 5′-TGCTGAAGTTTAGTCCAGTG-3′; hTMEM79 forward, 5′-CATCAAATGGGACTGTGGTG-3′ and reverse 5′-TTAAAGGTGGGAAGTTACAGG-3′; hALK forward, 5′-AAGCTGTACTGTCCCACCTAAC-3′ and reverse 5′-CATATTGGCAGTCAGCGAAGAG-3′; hFECH forward, 5′-AGTAGACTTTGAGTGACCGTCC-3′ and reverse 5′-AAAGAATTGAAGCAGGCCCTTG-3′; hUBN1 forward, 5′-AAATCAAGGTGAAGGAATCGTC-3′ and reverse 5′-TCCTGTTTGGTGATCTGAG-3′; hFBXW7 forward, 5′-GGATTGATGAACCATTGCAC-3′ and reverse 5′-ATGTTCTCAGACATTTGCCT-3′; hSLC43A2 forward, 5′-TTTGGTGGGATGTGTATGAC-3′ and reverse 5′-CATAGAAGAGCTTGATTCCTG-3′; hGUS forward, 5′-CTCATTTGGAATTTTGCCGAT-3′ and reverse 5′-CCGAGTGAAGATCCCCTTTTT-3′. hGUS was used as a normalization gene since its expression did not significantly fluctuate between groups in this study.

### Statistical Analysis

Data normalization was performed in 〈〈R〉〉 (http://www.r-project.org/index.html) and the bioconductor package limma. Two-color GenePix files were normalized within arrays using the “loess” method and between arrays with “Rquantile” normalization. Five groups of 9 samples were established and used to calculate p-values with respect to the analysis of variance (aov) within each group. Log-ratios were extracted for probes in which the aov p-value was less than or equal to 0.001, resulting in 1055 probes before unsupervised statistical analysis by principal component analysis (PCA) with SIMCA P^+^ software (Umetrics). Class prediction was done with the nearest centroid method using ClaNC software, which is known to make the classifier more accurate by reducing the effect of noisy genes [16]. Significance Analysis of Microarrays (SAM) computed by SAM 3.0 software which can compute the false discovery rate (FDR) for the differentially expressed genes was used as “power analysis” [34]. ROC curve analysis was performed using MedCalc software (www.medcalc.be). Continuous variables are presented as mean ± SD and categorical as numbers and percentages. Continuous variables were compared with the use of Student's *t*-test or Mann-Whitney rank sum test when normality or equal variance test failed. Categorical variables were compared with the use of the Pearson chi-square (sigma stat) otherwise specified in the text.

## Supporting Information

Figure S1 **Realtime qPCR analysis of white blood cell genes expression.** RF: cardiovascular risk factors patients; ALVD: asymptomatic left ventricular dysfunction individuals. Arbitrary units are indicated (A.U.) after normalisation to the reference gene. * p<0.05; **p<0.01; *** p<0.001 in Student's *t* test.
(TIF)

Figure S2 **Realtime qPCR analysis of genes expression in humant heart.** Cont: Control patients without heart failure; CHF: Chronic heart failure patients. Arbitrary units are indicated (A.U.) after normalisation to the reference gene. * p<0.05; **p<0.01; *** p<0.001 in Student's t test.
(TIF)
